# Dr. Alfred P. Southwick

**Published:** 1898-09

**Authors:** 


### DR. ALFRED P. SOUTHWICK.

Dr. Southwick died at his residence in the city of Buffalo, New York, June 11, 1898.

He was born in Ashtabula, Ohio, in 1826.

In early manhood he removed to the city of Buffalo, and became a steamboat engineer, and in 1855 was chosen chief engineer of the Western Transit Company. In 1862 he commenced the practice of dentistry, after some five years' preliminary training.

He was one of the organizers of the State Dental Society in 1868, and for twenty years was a member of the State Examining Board. He was one of the founders of the Dental Department of the University of Buffalo, and in this he has been an efficient coworker with his colleagues, his personal influence and ability having had much to do with the success attained by this college.

Dr. Southwick possessed a marked independence of character, which, combined with sterling honesty, raised him above the motives that influence the actions of many men. True to his own convictions of duty, he secured the respect of his professional associates.

His death will leave a vacant place difficult to fill, not alone in his immediate circle, but in that larger field of work where the influence of such men is needed more and more as the dental profession advances towards higher things.

